# The Preparation of Golgi Apparatus-Targeted Polymer Dots Encapsulated with Carbon Nanodots of Bright Near-Infrared Fluorescence for Long-Term Bioimaging

**DOI:** 10.3390/molecules28176366

**Published:** 2023-08-31

**Authors:** Yiping Lu, Wei Song, Zhiquan Tang, Wenru Shi, Shumei Gao, Jun Wu, Yuan Wang, Hu Pan, Yangang Wang, Hong Huang

**Affiliations:** 1 College of Biological, Chemical Science and Engineering, Jiaxing University, Jiaxing 314001, China00194156@zjxu.edu.cn (Z.T.);; 2 College of Advanced Materials Engineering, Jiaxing Nanhu University, Jiaxing 314001, China; 3 Institute for Agri-Food Standards and Testing Technology, Shanghai Academy of Agricultural Science, Shanghai 201403, China

**Keywords:** Golgi apparatus, carbon nanodots, polymer dots, near-infrared fluorescence, high photostability, long term

## Abstract

As a vital organelle in eukaryotic cells, the Golgi apparatus is responsible for processing and transporting proteins in cells. Precisely monitoring the status of the Golgi apparatus with targeted fluorescence imaging technology is of enormous importance but remains a dramatically challenging task. In this study, we demonstrate the construction of the first Golgi apparatus-targeted near-infrared (NIR) fluorescent nanoprobe, termed Golgi-Pdots. As a starting point of our investigation, hydrophobic carbon nanodots (CNDs) with bright NIR fluorescence at 674 nm (fluorescence quantum yield: 12.18%), a narrow emission band of 23 nm, and excellent stability were easily prepared from Magnolia Denudata flowers using an ultrasonic method. Incorporating the CNDs into a polymer matrix modified with Golgi-targeting molecules allowed for the production of the water-soluble Golgi-Pdots, which showed high colloidal stability and similar optical properties compared with pristine CNDs. Further studies revealed that the Golgi-Pdots showed good biocompatibility and Golgi apparatus-targeting capability. Based on these fascinating merits, utilizing Golgi-Pdots for the long-term tracking of the Golgi apparatus inside live cells was immensely successful.

## 1. Introduction

As one of the vital organelles in eukaryotic cells, the Golgi apparatus primarily participates in receiving, processing, and modifying diverse proteins generated by the endoplasmic reticulum, and then precisely packaging and delivering them to specific regions in the cell or secreting them outside it [1,2,3]. Since it is implicated in such important intracellular activities, subtle pH, viscosity, or morphological changes in the Golgi apparatus would interrupt the biosystem and affect cell survival, leading to various diseases, such as vascular disease, acute liver injury, hypertension, and cancer [4,5,6,7]. As such, it is highly significant to exploit specialized probes to the Golgi apparatus for the real-time visualization of its intracellular dynamics and to uncover the pathogenesis of Golgi-associated disease.

By virtue of its multiple intrinsic characteristics, such as superb sensitivity, high temporal resolution, real-time responsiveness, and noninvasiveness, fluorescence imaging has proved to be a powerful tool for the in situ dynamic tracking and visualizing of various organelles inside cells [8,9,10,11,12,13]. Despite some types of Golgi apparatus-targeted molecular probes, such as Golgi Tracker Green or Red derived from boron-dipyrromethene, having been produced commercially, their present status is still far from ideal as they often suffer from poor photostability at a working concentration [14]. Recently, Tsukiji and coworkers reported a series of Golgi-localizing fluorescent molecular probes by modularly linking tri-N-methylated myristoyl-Gly-Cys lipopeptide to fluorophores of interest [15]. Choi et al. synthesized yellow- and blue-emissive two-photon probes derived from 2,5-bis(benzo[d]oxazol-2-yl)pyrazine and 6-(benzo[d]oxazol-2-yl)-2-naphthalylamine derivatives, acting as the fluorescence moieties, and a trans-Golgi network peptide (SDYQRL), working as the Golgi apparatus-directing ligand, for Golgi apparatus detection [16]. Unfortunately, these probes were derived from organic fluorophores, which might also suffer from the photobleaching effect, restricting their further applications.

Carbon nanodots (CNDs), with the advantages of low-cost, tunable optical properties, excellent stability, and favorable biocompatibility, have received enormous research interest in the imaging of the Golgi apparatus and other organelles [17,18,19]. For example, Liu’s group produced two kinds of CNDs with blue emission, choosing D/L-penicillamine and citric acid as raw materials, for specifically the illuminating Golgi apparatus [20]. Zhang et al. synthesized sulfonamide-functionalized CNDs with a red emission peak at 612 nm for the targeted imaging of the Golgi apparatus [6]. Wei and coworkers reported a one-step hydrothermal method with ascorbic acid as the carbon raw source and L-cysteine as the targeting moiety to prepare blue-emitting carbon nanodots for the selective labeling of the Golgi apparatus [21]. The fluorescence signals of all the above Golgi apparatus-targeted CNDs are located in the visible light spectrum of 400 to 650 nm, which will be influenced by the autofluorescence originating from endogenous substances, such as reticulin, flavins, and reduced nicotinamide adenine dinucleotide (NADH), that are also located in this wavelength region [22]. In comparison to the fluorescence located in the visible region, near-infrared (NIR) fluorescence, in the wavelength range between 650 and 900 nm, displays relatively low autofluorescence and enables the high-sensitivity monitoring of active molecules and biological processes [23]. However, to the best of our knowledge, a Golgi apparatus-targeted NIR fluorescent probe based on CNDs has not been reported to date.

**Scheme 1:**
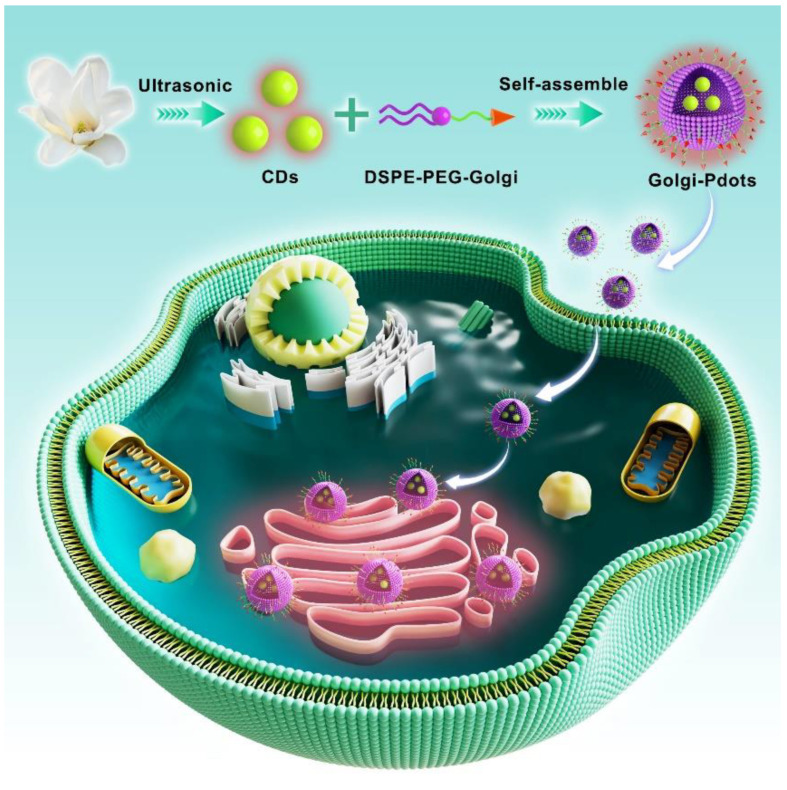
Schematic presentation of the synthesis of NIR-emissive CNDs and Golgi-Pdots and their application in Golgi apparatus-targeted imaging.

In this work, Golgi apparatus-targeted NIR fluorescent polymer dots (Golgi-Pdots) incorporated with NIR fluorescent hydrophobic CNDs were constructed for the specific and long-term bioimaging of the Golgi apparatus (Scheme 1). As a starting point of our investigation, hydrophobic CNDs with bright NIR fluorescence at 674 nm (fluorescence quantum yield: 12.18%), a narrow emission band of 23 nm, and excellent stability were easily prepared from Magnolia Denudata flowers through an ultrasonic-assisted method. Incorporating the CNDs into a polymer matrix modified with Golgi apparatus-targeting molecules allowed us to produce the water-soluble Golgi-Pdots, which showed high colloidal stability and similar optical properties to the CNDs. Further studies revealed that the Golgi-Pdots showed superior photostability, excellent biocompatibility, and Golgi apparatus-targeting capability. Based on these fascinating merits, utilizing Golgi-Pdots for the long-term tracking of the Golgi apparatus inside live cells was immensely successful.

## 2. Results and Discussions

### 2.1. Preparation and Characterization of NIR-Emissive CNDs

**Figure 1:**
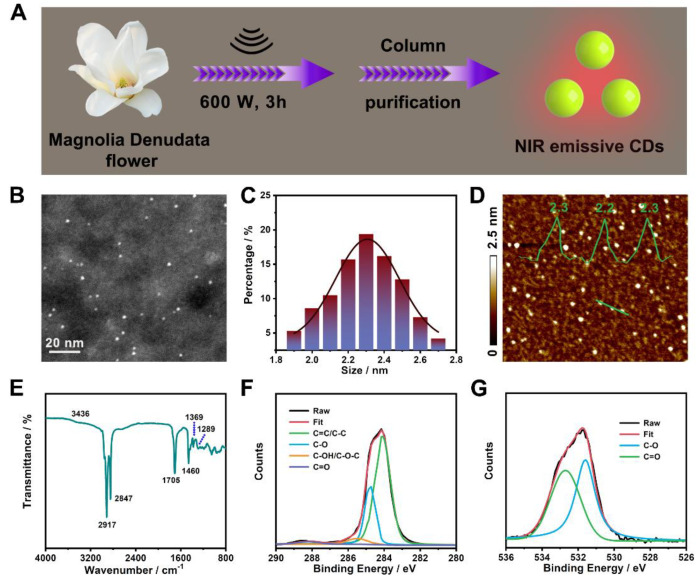
(**A**) Schematic presentation of the fabrication of NIR-emissive CNDs. (**B**) STEM image of CNDs. (**C**) Size distribution of CNDs. (**D**) AFM image of CNDs. Inset: the corresponding height profile analysis along the green line. (**E**) FTIR spectrum of CNDs. (**F**) High-resolution C1s spectrum. (**G**) High-resolution O1s spectrum.

As illustrated in Figure 1A, NIR-emissive CNDs can be simply prepared at room temperature from Magnolia Denudata flowers through an ultrasonic method. It is worth noting that the ultrasonic technique has recently become an immensely popular tool for nanomaterial preparation because of its appealing merits, such as rapid reaction rate, ease of operation, and controllable reaction conditions [24,25,26,27,28,29]. Ultrasonic waves generate acoustic cavitation within an aqueous solution, and this is the main reason for the creation, growth, and collapse of microbubbles [30]. During the above process, an instantaneous high-temperature (>5000 K) and high-pressure environment (>20 MPa) is created [31], which would facilitate carbohydrates in Magnolia Denudata flowers to undergo dehydration, decomposition, polymerization, and/or aromatization to form CNDs via nucleation [32,33]. The as-generated CNDs were purified via silica gel column chromatography to wipe off impurities.

The particle size and morphology of the CNDs were investigated using STEM and AFM. From the STEM image depicted in Figure 1B, we can see that the dot-shaped CNDs are wildly and uniformly distributed, with a sharp particle size distribution in the range from 1.9 to 2.7 nm and a mean value of 2.3 nm (Figure 1C), as calculated from about 150 individual nanodots. This size is comparable to other biomass-derived NIR fluorescent CNDs [34,35], and no larger nanoparticles are noticeable, revealing that the established methodology had good control of size. The AFM image in Figure 1D shows that the CNDs are evenly distributed, showing a height of 2.3 nm (inset of Figure 1D), which is in good agreement with STEM data.

To analyze the functional groups on the surfaces of the CNDs, an FTIR spectroscopy experiment was conducted. As shown in the FTIR spectrum (Figure 1E), the absorption band at 3436 cm^−1^ arises from the -OH group [36,37], and the strong peaks at 2917 and 2847 cm^−1^ can be attributed to the -CH_3_ and -CH_2_ stretching vibrations, respectively [38,39]. Moreover, the peaks at 1705, 1460, 1369, and 1289 cm^−1^ are ascribed to the stretching vibrations of the C=O, C=C, symmetric carboxylate, and C-O groups, respectively [31,40,41,42,43].

Meanwhile, XPS spectroscopy was also applied to characterize the chemical composition of the CNDs. According to the XPS survey result (Figure S1), only C1s (284.4 eV) and O1s (531.9 eV) signals could be observed [44], suggesting the CNDs were comprised of C (77.1%) and O (22.9%). The high-resolution XPS spectra of C1s and O1s were deconvoluted for further dissection. Those of C1s (Figure 1F) exhibit four peaks at 284.1, 284.7, 285.5, and 288.2 eV, assigned to the presence of C=C/C-C, C-O, C-O-C/C-OH, and C=O bonds, respectively [45,46,47]. The O1s band was deconvoluted into two typical peaks at 531.6 and 532.7 eV (Figure 1G), which are attributed to C-OH/C-O-C and C=O, respectively [48,49,50]. Together, these results suggest the existence of ether, carboxyl, amide, and hydrocarbon groups (-CH_2_ and -CH_3_) on the surface of the CNDs.

**Figure 2:**
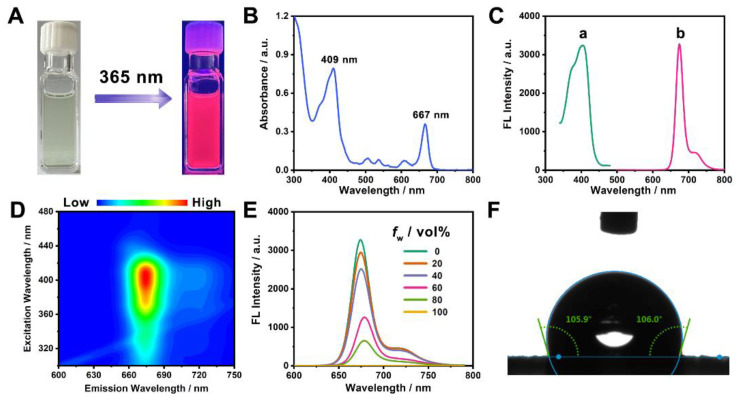
(**A**) Ethanol solution of CNDs under (left) natural light and (right) 365 nm UV light. (**B**) Absorption spectrum of CNDs. (**C**) Fluorescence (a) excitation and (b) emission spectrum of CNDs. (**D**) Excitation–emission contour plot of the CNDs in ethanol. (**E**) Fluorescence emission spectra of CNDs in ethanol–water mixed solutions with various water fractions (*f_w_*). (**F**) Contact angle of water on the CD film.

Subsequently, the optical features of the CNDs in ethanol solution were explored with UV–visible absorption and fluorescence spectroscopy. This solution showed a clear yellowish-green color in the room’s light and emitted bright red fluorescence under 365 nm UV light (Figure 2A). As can be seen from the absorption spectrum in Figure 2B, the CNDs had a very broad absorption band covering the UV to the visible regions, with two intense absorption peaks at 409 and 667 nm, which possibly stem from the n-π* transitions of aromatic C=O structures [51].

The fluorescence spectra plotted in Figure 2C show that the CNDs exhibited a fluorescence emission peak at 674 nm when excited under the maximum excitation wavelength of 406 nm, with a large Stokes shift up to 268 nm and a full width at the half-maximum (FWHM) of 23 nm. Such a narrow near-infrared emission is very close to that of CNDs derived from taxus leaves [34], but our preparation procedure is time-saving and, thus, has high efficiency. The excitation–emission contour plot of the CNDs shows that their emission center was actually unchanged over a wide excitation range, revealing the excitation-independent emission character of the CNDs (Figure 2D). In addition, their absolute quantum yield in ethanol was measured to be 12.18%, demonstrating their strong potential for application in NIR imaging. In addition, we unveiled that the CNDs displayed intense NIR fluorescence in various organic solvents of different polarities (Figure S2). However, when introducing water into the organic solution of the CNDs, their fluorescence intensity progressively weakened and eventually became nonemissive, with the water proportion increasing from 0% to 100% (volume ratio), suggesting our obtained CNDs had a hydrophobic character (Figure 2E). This result was confirmed by using a water contact angle experiment, which showed a static contact angle of 106.0° (Figure 2F). The hydrophobicity of the CNDs was due to the fact that hydrophobic long alkyl chains existed on the surface, as verified via XPS and FTIR characterizations.

### 2.2. Fabrication and Characterization of Pdots Incorporated with CNDs

**Figure 3:**
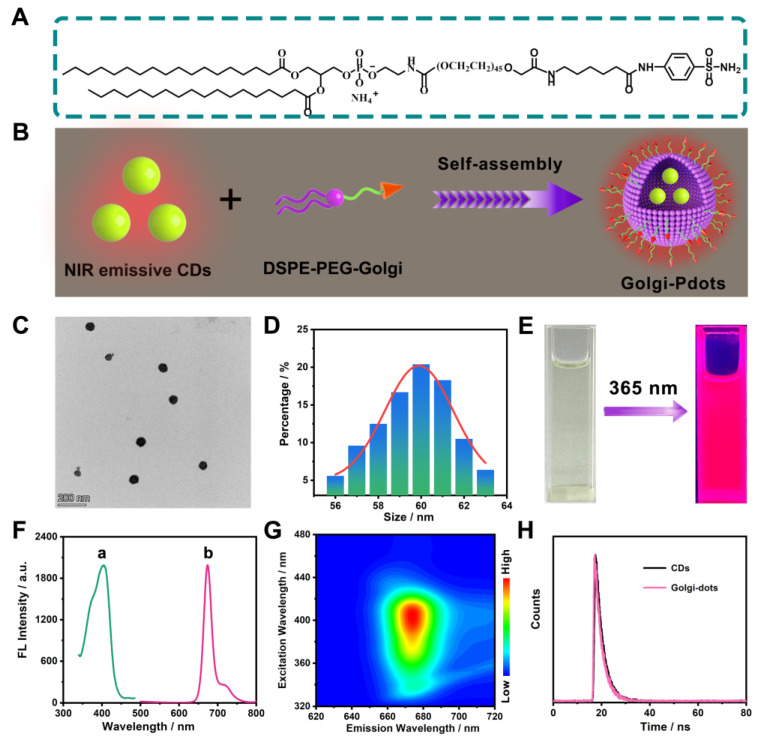
(**A**) Chemical structure of DSPE-PEG-Golgi. (**B**) Schematic presentation of the synthesis of NIR fluorescent Golgi-Pdots. (**C**) TEM image of Golgi-Pdots. (**D**) Size distribution of Golgi-Pdots. (**E**) The PBS solution of Golgi-Pdots under (left) natural light and (right) 365 nm UV light. (**F**) Fluorescence spectra of Golgi-Pdots ((a) excitation spectrum; (b) emission spectrum). (**G**) Excitation–emission contour plot of Golgi-Pdots in PBS solution. (**H**) Fluorescence decay curve of (black curve) CNDs and (pink curve) Golgi-Pdots under excitation of 406 nm. The fluorescence lifetime curves were well fitted to a single exponential decay.

In order to realize the potential applications of the hydrophobic CNDs with bright near-infrared fluorescence in a biosystem, nanoprecipitation experiments were carried out to shift the CNDs from the organic phase to the aqueous phase with the assistance of 1,2-distearoyl-sn-glycero-3-phosphoethanolamine-N-(polyethylene glycol)-2000 (DSPE-PEG), an amphiphilic polymer. Because of their high biocompatibility, DSPE-PEG derivatives are widely utilized as a matrix to encapsulate hydrophobic components to afford water-soluble nanoparticles with tiny size (<100 nm) and good colloidal stability [52,53,54,55,56]. As reported in the literature [6,57], SC-558 is an inhibitor of the Golgi protein Cyclooxygenase-2, and the corresponding functional fragment is a phenylsulfonamide moiety with the ability to bind with the active pocket site of Cyclooxygenase-2 via hydrogen bonds. Here, to endow the DSPE-PEG-assembled nanoparticles (Golgi-Pdots) with Golgi apparatus-targeting ability, DSPE-PEG-Golgi—prepared from DSPE-PEG with a terminal carboxyl group (DSPE-PEG-COOH) and phenylsulfonamide, the targeting moiety—was used in the nanoprecipitation experiment (Figure 3A).

Our hydrophobic CNDs were incorporated into the DSPE-PEG-Golgi through a nanoprecipitation method to prepare water-soluble Golgi-Pdots (Figure 3B). The TEM image in Figure 3C reveals that the generated Golgi-Pdots were quasi-spherical spheres and monodispersed with an average particle size of 60 nm (Figure 3D). Like the CNDs in ethanol, the Golgi-Pdots in PBS solution showed a yellowish-green color and emitted bright red fluorescence under 365 nm irradiation (Figure 3E). Fluorescence measurements demonstrated that the Golgi-Pdots in PBS solution also showed a maximum emission at 674 nm when being excited under 406 nm (Figure 3F), with a fluorescence quantum yield of 11.98%. Figure 3G shows the excitation-independent feature of the prepared Golgi-Pdots. Moreover, the fluorescence decay curves of the emission at 674 nm from the CNDs and Golgi-Pdots were fitted, showing a single decay time of 3.01 and 2.94 ns, respectively (Figure 3H). These experimental data demonstrate that encapsulating the CNDs into the polymer matrix showed almost no adverse effect on its optical properties.

**Figure 4:**
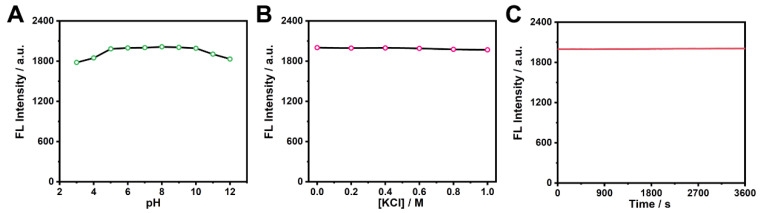
(**A**) Influences of pH values on the fluorescence intensities at 674 nm of Golgi-Pdots. (**B**) Influences of ionic strengths on the fluorescence intensities at 674 nm of Golgi-Pdots. (**C**) Dependence of fluorescence intensity at 674 nm on excitation time for Golgi-Pdots under 406 nm.

Next, the fluorescence stabilities of the Golgi-Pdots under different circumstances were examined. Figure 4A shows their fluorescence intensities at various pH values. There were no obvious variations in the fluorescence intensities from pH 5.0 to pH 10.0, which could make Golgi-Pdots usable in the physiological pH range. Their stability in a high-ionic solution using KCl salt was also studied. As shown in Figure 4B, the fluorescence intensity of the Golgi-Pdots remained unchanged with increasing KCl concentrations up to 1 M, demonstrating their great stability in high-ionic-strength environments. Photostability is another critical feature for probes to be applied in long-term bioimaging. In this regard, the photostability of the Golgi-Pdots in PBS solution was explored. As depicted in Figure 4C, they possessed excellent photostability, as nearly no photobleaching of the fluorescence intensity at 674 nm was noticeable after being irradiated continuously at 406 nm for 1 h. All the above data demonstrate that the Golgi-Pdots held a robust fluorescence, which is significantly beneficial for their future practical applications.

### 2.3. Cellular Imaging

**Figure 5:**
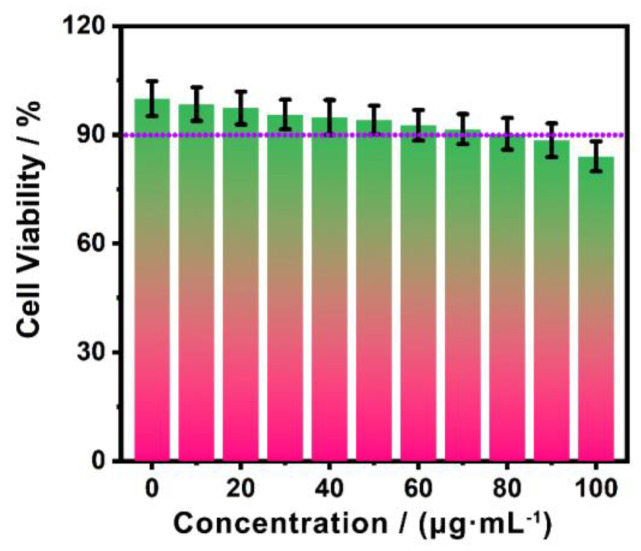
MTT test of HeLa cells cultivated with Golgi-Pdots of varied concentrations from 0 to 100 μg·mL^−1^ for 48 h.

The inherent toxicity of Golgi-Pdots to living cells was evaluated using a standard MTT test. As illustrated in Figure 5, the HeLa cells still showed viability values greater than 90% after exposure to Golgi-Pdots of a high concentration (up to 80 μg·mL^−1^) for 48 h. This result indicates the as-prepared Golgi-Pdots are biocompatible for use in the biological field.

**Figure 6:**
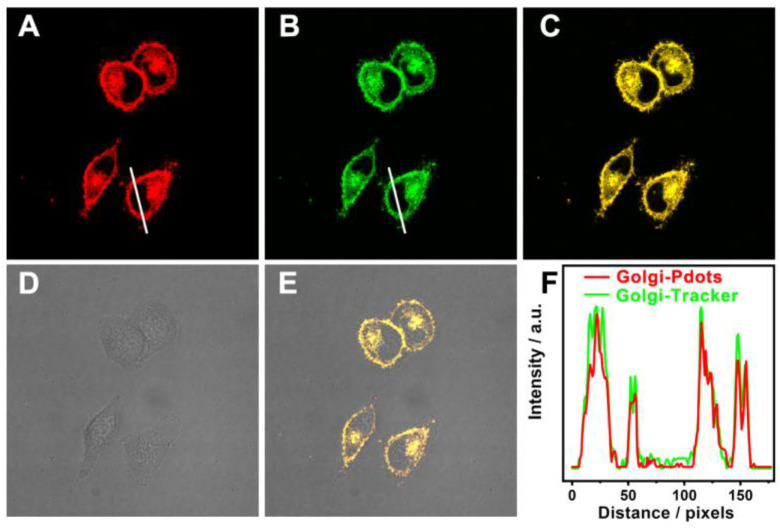
Subcellular localization studies in HeLa cells that were stained with Golgi-Pdots (10 μg mL^−1^) and Golgi-Tracker Green (50 nM) in order. (**A**) Red fluorescence of Golgi-Pdots (λ_ex_ = 405 nm; λ_em_ = 630–700 nm); (**B**) green fluorescence of Golgi-Tracker Green (λ_ex_ = 488 nm; λ_em_ = 500–570 nm); (**C**) the overlayed image of (**A**,**B**); (**D**) bright field image; (**E**) the overlayed image of (**C**,**D**); (**F**) intensity profiles of Golgi-Pdots and Golgi-Tracker Green along the white line in panel (**A**,**B**) across the HeLa cell.

Using the Golgi-Pdots as a fluorescent sensor, bioimaging investigations were conducted in vitro using confocal laser fluorescence microscopy. Considering the surface of the Golgi-Pdots was functionalized with a Golgi apparatus-targeting ligand, the probability of Golgi-Pdots specifically entering the Golgi apparatus of live cells was assessed. For this reason, colocalization tests were carried out on HeLa cells by costaining the Golgi-Pdots with commercial Golgi apparatus-specific dye, Golgi-Tracker Green, in order. Predictably, a bright red fluorescence signal was seen from Golgi-Pdots-labeled cells (Figure 6A), which overlapped very well with the green fluorescence of Golgi-Tracker Green (Figure 6B), as readily observed from the bright-yellow signals in Figure 6C (the merged image). Of note, the variations in the intensity profiles of the linear region of interest (the white line in Figure 6A,B) are synchronous in the two channels (Figure 6F). This result vividly establishes the fact that Golgi-Pdots can localize to the Golgi apparatus with high specificity.

**Figure 7:**
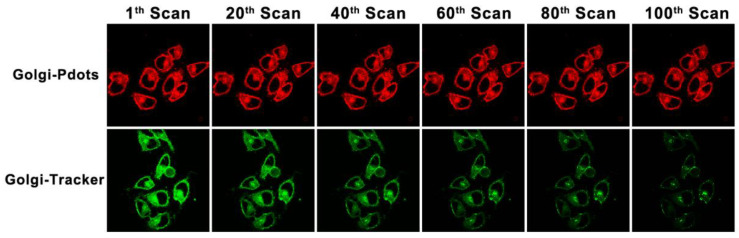
Photostability studies on Golgi-Pdots and Golgi-Tracker Green in cells. Fluorescence images of HeLa cells labeled with Golgi-Pdots (λ_em_ = 630–700 nm) and ER-Tracker Green (λ_em_ = 500–570 nm) after being scanned for varying numbers of times.

Moreover, in contrast with the commercial Golgi-Tracker Green, Golgi-Pdots are far more tolerant towards photobleaching. As can be found in Figure 7, the green fluorescence of Golgi-Tracker Green was nearly totally quenched at the 100th scan, whereas the red fluorescence from Golgi-Pdots saw no significant change. Notably, the fluorescence of Golgi-Pdots remained bright and stable during 1 h of continuous observation (Figure S3). These observations affirm that Golgi-Pdots can selectively enter the Golgi apparatus and be utilized for its long-term tracking in live cells.

## 3. Experimental Section

### 3.1. Materials

Magnolia Denudata flowers were purchased from a local supermarket. Before use, these flowers were rinsed carefully with deionized water and then dehydrated. 1,2-distearoyl-sn-glycero-3-phosphoethanolamine-N-(polyethylene glycol)-2000 (DSPE-PEG) modified with phenylsulfonamide was received from Hangzhou Xinqiao Biotechnology Co., Ltd. (Hangzhou, China). Acetone was purchased from Sinopharm Chemical Reagent Co., Ltd. (Shanghai, China). KCl and organic solvents, including petroleum ether, DCM, n-butanol, ethyl acetate, dioxane, acetonitrile, methanol, and dimethyl sulfoxide, were obtained from Shanghai Titan Technology Co., Ltd. (Shanghai, China). Golgi-Tracker Green was bought from Beyotime Biotech (Shanghai, China). High-glucose Dulbecco’s modified Eagle’s media (DMEM), 1× phosphate-buffered saline (PBS), and human cervical carcinoma cells (HeLa cells) were obtained from KeyGEN Biotech. Co., Ltd. (Nanjing, China) 3-(4,5-Dimethylthia-zol-2-yl)-2,5-diphenyltetrazolium bromide (MTT) was received from Sigma-Aldrich (St. Louis, MI, USA). All reagents and solvents were of analytical grade and used directly.

### 3.2. Characterizations

The absorption spectra were recorded on a Shimadzu UV-2550 spectrophotometer. The fluorescence excitation and emission spectra were measured on a Hitachi F-4600 fluorometer (excitation slit: 10 nm; emission slit: 10 nm; scan speed: 1200 nm min^−1^; and voltage: 500 V). The quantum yields and fluorescence lifetimes were analyzed with time-correlated single-photon counting on an FLS920 spectrometer. Scanning transmission electron microscopy (STEM) and transmission electron microscopy (TEM) characterizations were performed on a Thermoscientific Talos F200X transmission microscope working at 200 kV. Atomic force microscopic (AFM) experiments were performed in the ScanAsyst mode under ambient conditions (Bruker). X-ray photoelectron spectroscopy (XPS) experimental results were acquired utilizing a thermoelectron instrument (ESCALAB250, Thermo Fisher Scientific, MA, USA). Fourier-transform infrared spectroscopy (FTIR) spectra of the samples were collected using a Nicolet iS10 FTIR spectrometer. The contact angles of water on a film of CNDs were measured on a JC2000D contact angle goniometer (Shanghai Powereach Digital Technology Equipment Co., Ltd., Shanghai, China). Confocal fluorescence images of HeLa cells (512 × 512 pixels) were taken on a Leica TCS-SP8 confocal laser scanning microscope. A 63× objective lens was used for collecting cell images.

### 3.3. Preparation of NIR Fluorescent CNDs

CNDs with NIR fluorescence were fabricated via a facile, simple ultrasonic method [58]. In a typical synthesis, 20 g of dried flower of Magnolia Denudata and 800 mL of dichloromethane (DCM) were introduced in a 1000 mL flask, and an ultrasonic disposal experiment was conducted with a sonicator at 600 W output for 3 h (Scientz-IID, Ningbo Scientz Biotechnology Co., Ltd., Ningbo, China) using a pulse mode (10 s ON/10 s OFF). Then, the resulting clear-yellow solution was filtered through a syringe-driven filter with a pore size of 0.22 μm and centrifuged for 10 min at 12,000× *g* to discard large particles. The supernatant containing CNDs was collected and condensed, and the obtained crude product was further purified using a silica gel column (eluent: petroleum ether/ethyl acetate = 10:1, *v*/*v*) to afford the oily CNDs (0.18 g).

### 3.4. Fabrication of NIR-Emissive CND-Encapsulated Golgi-Pdots

NIR-emissive CNDs-encapsulated Golgi-Pdots were prepared using a nanoprecipitation method, according to the reported literature [59]. Briefly, 0.5 mg of CNDs and 2 mg of DSPE-PEG-Golgi were dissolved in 6 mL of DCM and gently stirred for 1 h at ambient temperature. Then, the solvent was evaporated under a nitrogen atmosphere and the product was dried in a vacuum oven. Following this, 6 mL of 1× PBS was quickly injected and stirred for another 1 h. The generated aqueous solution was filtrated with a polyethersulfone filter (pore size: 0.22 μm) and washed three times using centrifugal filter units (50 K) under centrifugation at 5000× *g* for 10 min. The obtained solution of Golgi-Pdots was lyophilized and the resulting lyophilizate was stored at 4 °C for future use.

### 3.5. Stability Tests

For investigating the influence of pH values on the fluorescence intensities of Golgi-Pdots, lyophilized Golgi-Pdots with a weight of 10 μg were dissolved in 1 mL PBS solutions with pH values varying from 3.0 to 12.0. The fluorescence intensities of the solutions were then measured under the excitation of 406 nm.

Similarly, for studying the influences of ionic strengths on the fluorescence intensities of Golgi-Pdots, lyophilized Golgi-Pdots with a weight of 10 μg were dissolved in 1 mL KCl solutions of concentrations varying from 0 to 1 M KCl. Again, the fluorescence intensities of the solutions were then measured under the excitation of 406 nm.

For assessing the photostability of the Golgi-Pdots, 2 mL of Golgi-Pdot solution with a concentration of 10 μg mL^−1^ was put in a 3 mL quartz cuvette with a length of 1 cm. Then, the fluorescence intensity at 674 nm of the Golgi-Pdots was continuously collected under the excitation of 406 nm.

### 3.6. Cytotoxicity Assessment

HeLa cells with a density of ~1 × 10^4^ cells per well were planted in 96-well plates and cultivated in DMEM supplemented with 10% fetal bovine serum, 80 μg mL^−1^ streptomycin, and 80 U mL^−1^ penicillin in a humid atmosphere with 95% air/5% CO_2_. Then, 12 h later, the cultivating medium was changed to a new one containing Golgi-Pdots of different concentrations (0–100 μg mL^−1^) and cultivated for another 48 h. As for each concentration, five independent experiments were performed. Following this, 20 μL of MTT solution (1.0 mg mL^–1^) was introduced into the wells, and then cultivated for another 4 h to promote the generation of formazan crystals. Subsequently, 150 μL of DMSO was injected into each well. The absorbance intensity, abbreviated as A, of the resultant mixture was determined at 570 nm. Cellular viabilities were quantified based on the following equation: cellular viability (%) = A_test_/A_control_ × 100%, where A_control_ stands for the absorbance value obtained from the control group, and A_test_ stands for the absorbance recorded with the existence of Golgi-Pdots.

### 3.7. Fluorescent Imaging

Before performing bioimaging experiments, HeLa cells were passaged, replanted into a confocal dish with 4 wells, and allowed to adhere for about 12 h. Soon afterwards, the growth media in the 4-well dishes were substituted with new ones that contained Golgi-Pdots (10 μg·mL^−1^) and incubated for another 1 h. Subsequently, the attached cells on the dishes were rinsed thrice with pure DMEM. The confocal fluorescence imaging of Golgi-Pdot-labeled cells was collected in the wavelength region from 630 to 700 nm (excitation wavelength: 405 nm).

The subcellular location of Golgi-Pdots was assessed using colocalization bioimaging tests, in which HeLa cells were sequentially labeled with Golgi-Pdots (10 μg·mL^−1^) for 1 h and Golgi-Tracker Green (500 nM) for 0.5 h [60]. Afterwards, the labeled cells were washed three times with pure DMEM and then subjected to confocal imaging. The fluorescence signal of the Golgi-Pdots was acquired in the wavelength region of 630–700 nm (excitation wavelength: 405 nm), while for Golgi-Tracker Green, fluorescence emission signals were acquired between 500 and 570 nm (excitation wavelength: 488 nm).

## 4. Conclusions

In conclusion, hydrophobic CNDs with bright NIR fluorescence and a narrow emission band were prepared from Magnolia Denudata flowers via an ultrasonic-assisted method and purified via silica gel column chromatography. To endow the CNDs with good water dispersibility and Golgi-targeting ability, we encapsulated them into a polymer matrix to fabricate the desired water-soluble polymer dots, Golgi-Pdots. They showed high colloidal stability and similar optical properties compared to CNDs. Moreover, due to the existence of PEG and Golgi-targeting molecules on the surface, the Golgi-Pdots showed excellent biocompatibility and can be applied for the long-term bioimaging of the Golgi apparatus inside living cells. This work could provide a novel methodology for the synthesis of Golgi apparatus-targetable near-infrared fluorescent probes and widen their promising application potential in precise diagnosis and therapy.

## Data Availability

Not applicable.

## Supplementary Materials

The following supporting information can be downloaded at: https://www.mdpi.com/article/10.3390/molecules28176366/s1, Figure S1: XPS survey spectrum of the CNDs; Figure S2: Excitation–emission contour plots of the CNDs in different organic solvents; Figure S3: Chemical structure of DSPE-PEG-Golgi.

## Sample Availability

Samples of the compounds are available from the corresponding authors.

## References

[1] Chen J., Liu H., Yang L., Jiang J., Bi G., Zhang G., Li G., Chen X. (2019). Highly selective and efficient synthesis of 7-aminoquinolines and their applications as Golgi-localized probes. ACS Med. Chem. Lett..

[2] Liu M., Chen Y., Guo Y., Yuan H., Cui T., Yao S., Jin S., Fan H., Wang C., Xie R. (2022). Golgi apparatus-targeted aggregation-induced emission luminogens for effective cancer photodynamic therapy. Nat. Commun..

[3] Liu C., Zhu H., Zhang Y., Su M., Liu M., Zhang X., Wang X., Rong X., Wang K., Li X. (2022). Recent advances in Golgi-targeted small-molecule fluorescent probes. Coord. Chem. Rev..

[4] Zhu H., Liu C., Rong X., Zhang Y., Su M., Wang X., Liu M., Zhang X., Sheng W., Zhu B. (2022). A new isothiocyanate-based Golgi-targeting fluorescent probe for Cys and its bioimaging applications during the Golgi stress response. Bioorg. Chem..

[5] Wang H., He Z., Yang Y., Zhang J., Zhang W., Zhang W., Li P., Tang B. (2019). Ratiometric fluorescence imaging of Golgi H_2_O_2_ reveals a correlation between Golgi oxidative stress and hypertension. Chem. Sci..

[6] Zhang X., Chen L., Wei Y.-Y., Du J.-L., Yu S.-P., Liu X.-G., Liu W., Liu Y.-J., Yang Y.-Z., Li Q. (2022). Cyclooxygenase-2-targeting fluorescent carbon dots for the selective imaging of Golgi apparatus. Dye. Pigment..

[7] Liu C., Zhou L., Zheng Y., Man H., Ye Z., Zhang X., Xie L., Xiao Y. (2022). A Golgi-targeted viscosity rotor for monitoring early alcohol-induced liver injury. Chem. Commun..

[8] Chen B., Mao S., Sun Y., Sun L., Ding N., Li C., Zhou J. (2021). A mitochondria-targeted near-infrared fluorescent probe for imaging viscosity in living cells and a diabetic mice model. Chem. Commun..

[9] Zhang J., Han W., Zhou X., Zhang X., Zhang H., Li T., Wang J., Yuan Y., He Y., Zhou J. (2023). A lipid droplet-specific NIR fluorescent probe with a large stokes shift for in vivo visualization of polarity in contrast-induced acute kidney Injury. Anal. Chem..

[10] Liu Z., Tian Y. (2021). Recent advances in development of devices and probes for sensing and imaging in the brain. Sci. China Chem..

[11] Liu Z., Zhu Y., Zhang L., Jiang W., Liu Y., Tang Q., Cai X., Li J., LH W., Tao C. (2023). Structural and functional imaging of brains. Sci. China Chem..

[12] Gao P., Pan W., Li N., Tang B. (2019). Fluorescent probes for organelle-targeted bioactive species imaging. Chem. Sci..

[13] Huang H., Tian Y. (2018). A ratiometric fluorescent probe for bioimaging and biosensing of HBrO in mitochondria upon oxidative stress. Chem. Commun..

[14] Xiao P., Ma K., Kang M., Huang L., Wu Q., Song N., Ge J., Li D., Dong J., Wang L. (2021). An aggregation-induced emission platform for efficient Golgi apparatus and endoplasmic reticulum specific imaging. Chem. Sci..

[15] Sawada S., Yoshikawa M., Tsutsui K., Miyazaki T., Kano K., Mishiro-Sato E., Tsukiji S. (2023). Palmitoylation-dependent small-molecule fluorescent probes for live-cell Golgi imaging. ACS Chem. Biol..

[16] Choi J.-W., Hong S.T., Kim M.S., Paik K.C., Han M.S., Cho B.R. (2019). Two-photon probes for Golgi apparatus: Detection of Golgi apparatus in live tissue by two-photon microscopy. Anal. Chem..

[17] Paul S., Bhattacharya A., Hazra N., Gayen K., Sen P., Banerjee A. (2022). Yellow-emitting carbon dots for selective fluorescence imaging of lipid droplets in living cells. Langmuir.

[18] Tong L., Wang X., Chen Z., Liang Y., Yang Y., Gao W., Liu Z., Tang B. (2020). One-step fabrication of functional carbon dots with 90% fluorescence quantum yield for long-term lysosome imaging. Anal. Chem..

[19] Jiang L., Cai H., Zhou W., Li Z., Zhang L., Bi H. (2023). RNA-targeting carbon dots for live-cell imaging of granule dynamics. Adv. Mater..

[20] Yuan M., Guo Y., Wei J., Li J., Long T., Liu Z. (2017). Optically active blue-emitting carbon dots to specifically target the Golgi apparatus. RSC Adv..

[21] Wei Y., Gao Y., Chen L., Li Q., Du J., Wang D., Ren F., Liu X., Yang Y. (2023). Carbon dots based on targeting unit inheritance strategy for Golgi apparatus-targeting imaging. Front. Mater. Sci..

[22] Li J.-B., Liu H.-W., Fu T., Wang R., Zhang X.-B., Tan W. (2019). Recent progress in small-molecule near-IR probes for bioimaging. Trends Chem..

[23] Huang X., El-Sayed I.H., Qian W., El-Sayed M.A. (2006). Cancer cell imaging and photothermal therapy in the near-infrared region by using gold nanorods. J. Am. Chem. Soc..

[24] Zhang Y., Li K., Ren S., Dang Y., Liu G., Zhang R., Zhang K., Long X., Jia K. (2019). Coal-derived graphene quantum dots produced by ultrasonic physical tailoring and their capacity for Cu(II) detection. ACS Sustain. Chem. Eng..

[25] Huang H., Shen Z., Chen B., Wang X., Xia Q., Ge Z., Wang Y., Li X. (2020). Selenium-doped two-photon fluorescent carbon nanodots for in-situ free radical scavenging in mitochondria. J. Colloid Interf. Sci..

[26] Xu J., Cui K., Gong T., Zhang J., Zhai Z., Hou L., Zaman F.u., Yuan C. (2022). Ultrasonic-assisted synthesis of N-doped, multicolor carbon dots toward fluorescent inks, fluorescence sensors, and logic gate operations. Nanomaterials.

[27] Zhao W.-B., Liu K.-K., Song S.-Y., Zhou R., Shan C.-X. (2019). Fluorescent nano-biomass dots: Ultrasonic-assisted extraction and their application as nanoprobe for Fe^3+^ detection. Nanoscale Res. Lett..

[28] Kang J., Gao P., Zhang G., Shi L., Zhou Y., Wu J., Shuang S., Zhang Y. (2022). Rapid sonochemical synthesis of copper nanoclusters with red fluorescence for highly sensitive detection of silver ions. Microchem. J..

[29] Huang H., Li S., Chen B., Wang Y., Shen Z., Qiu M., Pan H., Wang W., Wang Y., Li X. (2022). Endoplasmic reticulum-targeted polymer dots encapsulated with ultrasonic synthesized near-infrared carbon nanodots and their application for in vivo monitoring of Cu^2+^. J. Colloid Interf. Sci..

[30] Gedanken A. (2004). Using sonochemistry for the fabrication of nanomaterials. Ultrason. Sonochem..

[31] Dang H., Huang L.-K., Zhang Y., Wang C.-F., Chen S. (2016). Large-scale ultrasonic fabrication of white fluorescent carbon dots. Ind. Eng. Chem. Res..

[32] He H., Zhang R., Zhang P., Wang P., Chen N., Qian B., Zhang L., Yu J., Dai B. (2023). Functional carbon from nature: Biomass-derived carbon materials and the recent progress of their applications. Adv. Sci..

[33] Yang X., Ai L., Yu J., Waterhouse G.I.N., Sui L., Ding J., Zhang B., Yong X., Lu S. (2022). Photoluminescence mechanisms of red-emissive carbon dots derived from non-conjugated molecules. Sci. Bull..

[34] Liu J., Geng Y., Li D., Yao H., Huo Z., Li Y., Zhang K., Zhu S., Wei H., Xu W. (2020). Deep red emissive carbonized polymer dots with unprecedented narrow full width at half maximum. Adv. Mater..

[35] Liu J., Kong T., Xiong H.-M. (2022). Mulberry-leaves-derived red-emissive carbon dots for feeding silkworms to produce brightly fluorescent silk. Adv. Mater..

[36] Zhao X., Li J., Liu D., Yang M., Wang W., Zhu S., Yang B. (2020). Self-enhanced carbonized polymer dots for selective visualization of lysosomes and real-time apoptosis monitoring. iScience.

[37] Wang B., Song H., Tang Z., Yang B., Lu S. (2022). Ethanol-derived white emissive carbon dots: The formation process investigation and multi-color/white LEDs preparation. Nano Res..

[38] Deng Y., Shen C., Zhao W., Zheng G., Jiao F., Lou Q., Liu K., Shan C.-X., Dong L. (2023). Biosynthesis of the narrowband deep-red emissive carbon nanodots from eggshells. ACS Sustain. Chem. Eng..

[39] Zhao W.-B., Wang R.-T., Liu K.-K., Du M.-R., Wang Y., Wang Y.-Q., Zhou R., Liang Y.-C., Ma R.-N., Sui L.-Z. (2022). Near-infrared carbon nanodots for effective identification and inactivation of Gram-positive bacteria. Nano Res..

[40] Zhou D., Huang H., Wang Y., Wang Y., Hu Z., Li X. (2019). A yellow-emissive carbon nanodot-based ratiometric fluorescent nanosensor for visualization of exogenous and endogenous hydroxyl radicals in the mitochondria of live cells. J. Mater. Chem. B.

[41] Zhao W.-B., Chen D.-D., Liu K.-K., Wang Y., Zhou R., Song S.-Y., Li F.-K., Sui L.-Z., Lou Q., Hou L. (2023). Near-infrared I/II emission and absorption carbon dots via constructing localized excited/charge transfer state for multiphoton imaging and photothermal therapy. Chem. Eng. J..

[42] Yu X.-W., Liu X., Jiang Y.-W., Li Y.-H., Gao G., Zhu Y.-X., Lin F., Wu F.-G. (2022). Rose bengal-derived ultrabright sulfur-doped carbon dots for fast discrimination between live and dead cells. Anal. Chem..

[43] Wang B., Wei Z., Sui L., Yu J., Zhang B., Wang X., Feng S., Song H., Yong X., Tian Y. (2022). Electron–phonon coupling-assisted universal red luminescence of o-phenylenediamine-based carbon dots. Light Sci. Appl..

[44] Zheng Y., Li X., Wei C., Gao Y., Han G., Zhao J., Zhang C., Zhang K., Zhang Z. (2023). Long-lived phosphorescent carbon dots as photosensitizers for total antioxidant capacity assay. Anal. Chem..

[45] Wang Z.-X., Hu L., Wang W.-J., Kong F.-Y., Wei M.-J., Fang H.-L., Li Q.-L., Wang W. (2022). One-pot green preparation of deep-ultraviolet and dual-emission carbon nanodots for dual-channel ratiometric determination of polyphenol in tea sample. Microchim. Acta.

[46] Liu K.-K., Song S.-Y., Sui L.-Z., Wu S.-X., Jing P.-T., Wang R.-Q., Li Q.-Y., Wu G.-R., Zhang Z.-Z., Yuan K.-J. (2019). Efficient red/near-infrared-emissive carbon nanodots with multiphoton excited upconversion fluorescence. Adv. Sci..

[47] Yang L., Huang H., Wang T., Zhou D., Chen Q., Li D., Chen S., Lin P. (2023). Endoplasmic reticulum-targetable selenium-doped carbon nanodots with redox-responsive fluorescence for in situ free-radical scavenging in cells and mice. Arab. J. Chem..

[48] Li L., Shi L., Jia J., Eltayeb O., Lu W., Tang Y., Dong C., Shuang S. (2020). Dual photoluminescence emission carbon dots for ratiometric fluorescent GSH sensing and cancer cell recognition. ACS Appl. Mater. Interfaces.

[49] Khan W.U., Qin L., Alam A., Zhou P., Peng Y., Wang Y. (2021). Water-soluble green-emitting carbon nanodots with enhanced thermal stability for biological applications. Nanoscale.

[50] Xu Y., Wang B., Zhang M., Zhang J., Li Y., Jia P., Zhang H., Duan L., Li Y., Li Y. (2022). Carbon dots as a potential therapeutic agent for the treatment of cancer-related anemia. Adv. Mater..

[51] Zhang L., Wang Y., Jia L., Zhang X., Xu J. (2023). Dynamic anti-counterfeiting and reversible multi-level encryption-decryption based on spirulina derived pH-responsive dual-emissive carbon dots. J. Lumin..

[52] Li K., Liu B. (2014). Polymer-encapsulated organic nanoparticles for fluorescence and photoacoustic imaging. Chem. Soc. Rev..

[53] Che W., Zhang L., Li Y., Zhu D., Xie Z., Li G., Zhang P., Su Z., Dou C., Tang B.Z. (2019). Ultrafast and noninvasive long-term bioimaging with highly stable red aggregation-induced emission nanoparticles. Anal. Chem..

[54] Wu Y., Han H.-H., He L., Li L., Zang Y., Li J., He X.-P., Ding Y., Cao W., James T.D. (2023). Selective detection of peroxynitrite using an isatin receptor and a naphthalimide fluorophore. Chem. Commun..

[55] Wang P., Wang Y., Xia X., Huang W., Yan D. (2023). Redox-responsive drug-inhibitor conjugate encapsulated in DSPE-PEG2k micelles for overcoming multidrug resistance to chemotherapy. Biomater. Sci..

[56] Lin Y.-X., Wang Y., An H.-W., Qi B., Wang J., Wang L., Shi J., Mei L., Wang H. (2019). Peptide-based autophagic gene and cisplatin co-delivery systems enable improved chemotherapy resistance. Nano Lett..

[57] Zhu H., Liu C., Liang C., Tian B., Zhang H., Zhang X., Sheng W., Yu Y., Huang S., Zhu B. (2020). A new phenylsulfonamide-based Golgi-targeting fluorescent probe for H_2_S and its bioimaging applications in living cells and zebrafish. Chem. Commun..

[58] Zhao W., Wang Y., Liu K., Zhou R., Shan C. (2022). Multicolor biomass based carbon nanodots for bacterial imaging. Chin. Chem. Lett..

[59] Long Z., Dai J., Hu Q., Wang Q., Zhen S., Zhao Z., Liu Z., Hu J.J., Lou X., Xia F. (2020). Nanococktail based on AIEgens and semiconducting polymers: A single laser excited image-guided dual photothermal therapy. Theranostics.

[60] Feng Z., Wu J., Jiang M., Sha J., Liu W., Ren H., Zhang W., Lee C.-S., Wang P. (2022). A rhodamine derivative-based fluorescent probe for visual monitoring of pH changes in the Golgi apparatus. Sens. Actuators B Chem..

