# Supplementary material for: The Preparation of Golgi Apparatus-Targeted Polymer Dots Encapsulated with Carbon Nanodots of Bright Near-Infrared Fluorescence for Long-Term Bioimaging
Source: Molecules. 2023 Aug 31;28(17):6366. doi: 10.3390/molecules28176366 (PMC10488926; doi:10.3390/molecules28176366)
Supplement: Supplementary file 1 [file molecules-28-06366-s001.zip › molecules-2560645-supplementary.pdf]

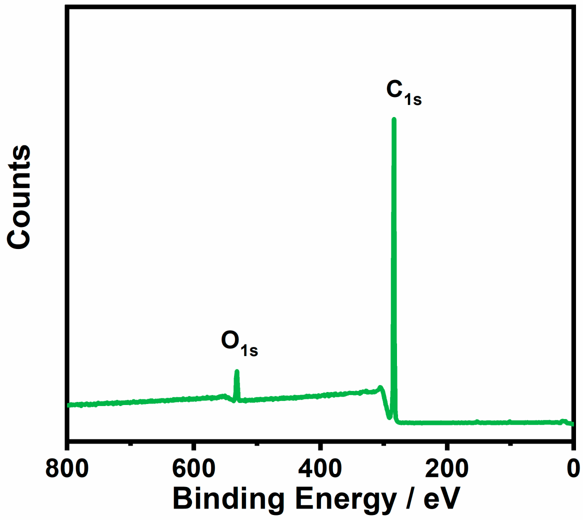

Figure S1 XPS survey spectrum of the CNDs.

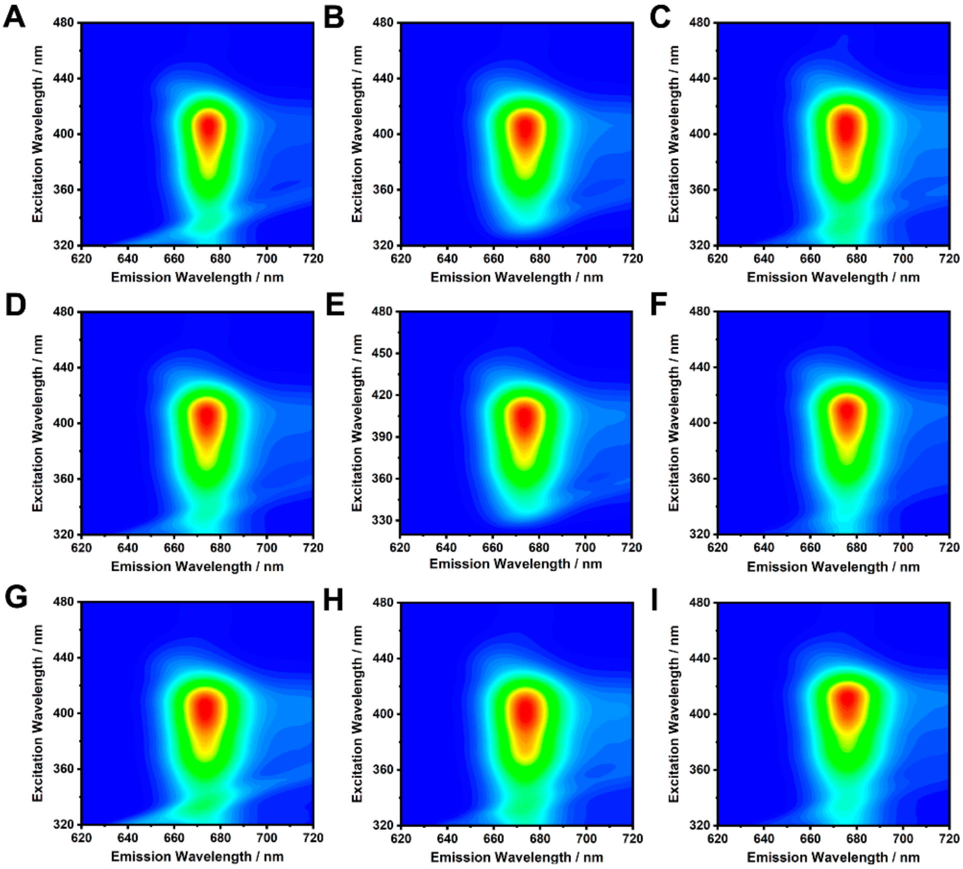

Figure S2 Excitation-emission contour plots of the CNDs in different organic solvents: (A) petroleum ether, (B) DCM, (C) n-butanol, (D) ethyl acetate, (E) dioxane, (F) acetone, (G) acetonitrile, (H) methanol, and (I) DMSO.

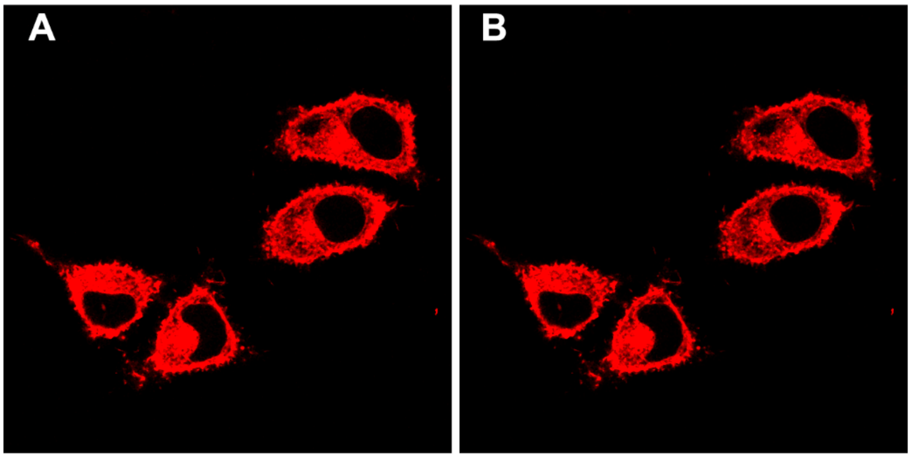

Figure S3 Temporal evolution of fluorescence signals of Golgi-Pdots in cells under continuous irradiation: (A) 0 min; (B) 60 min.
